# Raman Distributed Temperature Sensor with Optical Dynamic Difference Compensation and Visual Localization Technology for Tunnel Fire Detection

**DOI:** 10.3390/s19102320

**Published:** 2019-05-20

**Authors:** Baoqiang Yan, Jian Li, Mingjiang Zhang, Jianzhong Zhang, Lijun Qiao, Tao Wang

**Affiliations:** 1 College of Physics & Optoelectronics, Taiyuan University of Technology, Taiyuan, Shanxi 030024, China; yanbaoqiang0876@link.tyut.edu.cn (B.Y.); lijian0143@link.tyut.edu.cn (J.L.); zhangjianzhong@tyut.edu.cn (J.Z.); qiaolijun@tyut.edu.cn (L.Q.); wangmiaoshou@126.com (T.W.); 2 Key Laboratory of Advcanced Transducers and Intelligent Control System, Ministry of Education and Shanxi Province, Taiyuan, Shanxi 030024, China

**Keywords:** tunnel fire detection, Raman distributed fiber sensor, optical dynamic difference compensation, visual localization

## Abstract

The field of tunnel fire detection requires a Raman distributed temperature sensor (RDTS) with high-accuracy and visual localization. A novel temperature demodulation method to improve the temperature measurement accuracy of the RDTS systems is presented. This method is based on the optical dynamic difference compensation algorithm, which can eliminate the optical power fluctuation. In addition, the visual localization technology is presented by using the longitudinal lining model (LLM) of a three-dimensional (3D) temperature display, which enhances the engineering application of RDTS in tunnel fire detection. Experimental results indicate that the temperature measurement accuracy is optimized from 7.0 °C to 1.9 °C at the sensing distance of 18.27 km by using the presented method. We provide a solution for temperature field monitoring as well as fire visual localization of the tunnel through RDTS systems.

## 1. Introduction

A distributed optical fiber sensor for temperature monitoring has been invested in for the past thirty years due to its distinctive advantages of immune to electromagnetic interference, small size and resistance to ionizing radiation. It can operate safely in hazardous environment, with a fast response, corrosion resistance and is intrinsically safe [1,2,3,4]. The Raman distributed temperature sensor (RDTS) has been widely used in various infrastructures such as smart grids, oil well monitoring and fire alarm systems [5,6].

In 1985, the RDTS was first reported by Dakin et al. [7]. Over the past thirty years, a number of advanced techniques for improving the temperature measurement accuracy of RDTS systems have been proposed by researchers, which includes optical pulse coding techniques [8,9,10], a differential attenuation correction system [11], Rayleigh noise cancellation technology [12] a and constant temperature control system [13]. The optical pulse coding techniques improve the signal-to-noise (SNR) as well as the input optical power of RDTS systems. The differential attenuation correction system utilizes two light sources to cancel out the non-attenuation by Stokes and anti-Stokes light. The Rayleigh noise cancellation technology improves temperature measurement accuracy by eliminating the Rayleigh noise in Stokes and anti-Stokes light. The constant temperature control system optimizes temperature measurement accuracy by multi-stage thermostats. Moreover, to eliminate the influence of noise fluctuation on temperature measurement accuracy, some de-noising methods have been proposed and applied to the temperature demodulation algorithm, such as the wavelet modulus maximum de-noising algorithm [14,15], dynamic noise difference algorithm [16] and two-dimensional wavelet algorithm [17].

The application of RDTS for tunnel fire detection was proposed by Ishii et al. [18], but recently the critical issues of the RDTS systems have been an ambiguity in the backscattered intensity profile, which is the function of optical power affected by the instability of the resonant cavity or the changes of environment temperature [19,20]. The instability of optical power affects the temperature measurement accuracy of RDTS systems, which seriously limits the application of RDTS systems in tunnel engineering. Besides, the RDTS systems need to accomplish the pre-temperature calibration process before demodulating the temperature along the fiber [21,22,23]. Typically, it is considered that the optical power is constant during the calibration stage and the measurement stage [21,24]. Unfortunately, owing to the change of the operating current and environmental temperature, the optical power is inconsistent during the calibration stage and the measurement stage, which deteriorates the temperature measurement accuracy.

In addition, the temperature along the fiber is displayed as list or two-dimensional (2D) curve in conventional RDTS systems. For a large-scale data-monitoring systems, where the fiber distribution is complicated or the fiber is partially bent, this temperature display method cannot quickly locate the regions where temperature changes rapidly (such as local fire) into the actual three-dimensional (3D) environment. Kishida et al. proposed a 3D shape and displacement monitoring method by means of distributed optical fiber sensing for tunnel shape. The displacement is obtained and displayed in real time by the NeugreGate system. This method provides valuable information to the engineering team to help control the speed of the mining process and ensure its safety [25]. Soga et al. installed the cable in both longitudinal direction and cross section in a tunnel construction site in London, which not only achieves tunnel strain monitoring, but also clearly shows the mechanism of tunnel deformation that is useful for evaluating the engineering performance of tunnel construction. [26]. Tobias et al. employed fiber-optic sensors within Full-Scale Emplacement (FE) experiment to FE tunnel, obtained temperature distribution at FE tunnel wall by means of distributed temperature sensors. The proposed techniques yield good results from a full-scale demonstration experiment [27]. Sun et al. installed a part of two parallel fibers on the ceiling to locate the fire source in the room [28]. The method proposed above provides a good reference for the layout of optical cables in the tunnel and also realizes the health monitoring of the tunnel.

In this paper, a novel temperature demodulation method based on optical dynamic difference compensation is proposed to improve the temperature measurement accuracy for RDTS systems. Experimental results indicate that this novel temperature demodulation method can optimize the temperature measurement accuracy from 7.0 °C to 1.9 °C at the sensing distance of 18.27 km for multimode fiber. In addition, the visual localization technology is presented by using the longitudinal lining model (LLM) of a three-dimensional (3D) temperature display. This technology can meet visual localization for temperature rapid change regions of complex fiber distribution. The experimental and simulation results indicate that these studies provide a reliable solution for tunnel fire detection.

## 2. Theoretical Analysis

### 2.1. Optical Dynamic Difference Compensation Algorithm

The Raman scattering is caused by the irregular collision between the incident photon and the dielectric molecules in the fiber, generating anti-Stokes light with a frequency greater than the incident light and Stokes light having a frequency smaller than the incident light. Anti-Stokes light is temperature sensitive while Stokes light is temperature insensitive [5]. For RDTS systems, there are three temperature demodulation methods, which are a self-demodulation algorithm based on anti-Stokes light only, and a dual-demodulation algorithm based on the ratio of anti-Stokes light and Stokes light or the Rayleigh light [5]. Typically, in RDTS systems, the temperature information is extracted from the ratio of anti-Stokes light and Stokes light to eliminate the errors caused by factors including intensity fluctuation of the incident light, fiber attenuation, variation of the fiber core composition, and changes of the Raman capture coefficient along the fiber [29]. This temperature demodulation algorithm is widely used in RDTS systems to demodulate temperature information.

The self-demodulation algorithm demodulates the temperature along the fiber using the anti-Stokes light only; this method has no differential attenuation effect, with stronger signal-to-noise ratio compared to the dual-demodulation algorithm [30]. Assuming that the optical power and fiber attenuation remains constant throughout the temperature measurement process. The calibration stage is completed when the sensing fiber is maintained at a known temperature $T_{0}$, then the intensity of anti-Stokes Raman backscattered light at the point of *L* in the sensing fiber can be expressed as:

(1) $$F(T_{0},L)=K_{a}\cdot \nu_{a}^{4}\cdot S\cdot P\cdot \exp[-(\alpha_{0}+\alpha_{a})L]\cdot R_{a}(T_{0})$$

where $K_{a}$ is the coefficients related to the anti-Stokes Raman backscattered cross section, $\nu_{a}^{}$ is the frequency of the anti-Stokes Raman backscattered light, *S* is the backscatter factor of the fiber, *P* is the incident power,$\alpha_{0}$ and $\alpha_{a}$ is the average propagation loss of the incident light and the anti-Stokes light, respectively. $R_{a}\left(T_{0}\right)$ is the coefficient related with the population of lower and upper molecular energy level, which is dependent on the local domain temperature of the fiber.

In the temperature measurement stage, the intensity of anti-Stokes Raman backscattered light at the point of *L* can be expressed as:

(2) $$F(T,L)=K_{a}\cdot \nu_{a}^{4}\cdot S\cdot P\cdot \exp[-(\alpha_{0}+\alpha_{a})L]\cdot R_{a}(T)$$

By solving Equations (1) and (2), the temperature at the point of *L* can be demodulated:

(3) $$T\left(L\right)=\frac{h\Delta \nu /k}{\ln\left\{1+\left[F\left(T_{0},L\right)/F\left(T,L\right)\right]\left[\exp\left(h\Delta \nu /kT_{0}\right)-1\right]\right\}}$$

Actually, due to changes in environmental temperature, the mechanical structure of the resonator is unstable or the optical power fluctuates, etc. [18,19], resulting in optical power that is not constant during the calibration stage and measurement stage. The real intensity of anti-Stokes Raman backscattered light in the calibration stage and measurement stage can be expressed as:

(4) $$F(T_{0},L)=K_{a}\cdot \nu_{a}^{4}\cdot S\cdot P_{0}\cdot \exp[-(\alpha_{0}+\alpha_{a})L]\cdot R_{a}(T_{0})$$

(5) $$F(T,L)=K_{a}\cdot \nu_{a}^{4}\cdot S\cdot P_{1}\cdot \exp[-(\alpha_{0}+\alpha_{a})L]\cdot R_{a}(T)$$

where $P_{0}$ and $P_{1}$ represent the optical power in calibration stage and measurement stage, respectively. By solving Equations (4) and (5), the temperature at the point of L can be demodulated:

(6) $$T\left(L\right)=\frac{h\Delta \nu /k}{\ln\left\{1+\left(P_{1}/P_{0}\right)\left[F\left(T_{0},L\right)/F\left(T,L\right)\right]\left[\exp\left(h\Delta \nu /kT_{0}\right)-1\right]\right\}}$$

From Equation (6), it can be seen that the demodulated temperature is not only relates to the Raman scattering light $F(T_{0},L)$ and F(T,L), but also relates to the optical power ($P_{0}$ and $P_{1}$). If the optical power is inconsistent during the calibration stage and measurement stage ($P_{0}\neq P_{1}$), it will cause inaccurate temperature measurement.

**Figure 1:**
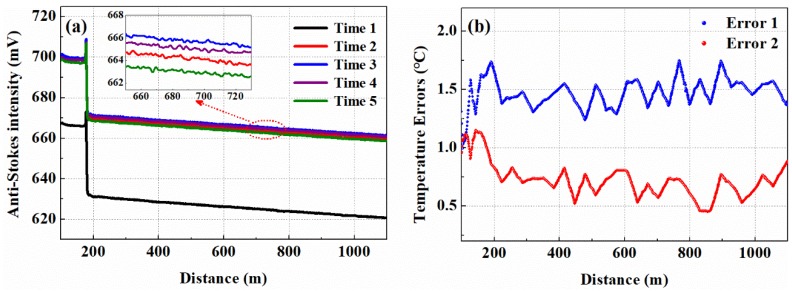
(**a**) The intensity of anti-Stokes backscattered light at different measuring times; (**b**) The temperature errors caused by different measuring times.

The intensity of anti-Stokes backscattered light at different times also explains this phenomenon. At room temperature, around 24 °C, the anti-Stoke light is collected at Time 1 in the calibration stage. In the measurement stage, the anti-Stoke light is collected at Time 2, Time 3, Time 4, Time 5, and the acquisition interval in the measurement stage is 90 s. Figure 1a shows the intensity of the anti-Stokes light exhibiting different states at different measuring times. Figure 1b shows the temperature-measurement errors caused by different measuring times. The blue curve represents the temperature-measurement error demodulated at Time 2 and Time 3, and the red curve represents the temperature-measurement error demodulated at Time 4 and Time 5. It can be seen from Figure 1 that under different measuring times, the fluctuation of optical power causes the intensity of anti-Stokes light exhibits different states, which leads to temperature-measurement error. In actuality, the intensity of the anti-Stokes backscattered light may be related to the optical power, the optoelectronic gain of avalanche photodiode (APD), and noise fluctuations of the sensing fiber.

In order to eliminate the temperature-measurement errors caused by optical power fluctuation, optical dynamic difference compensation is proposed. The proposed method is based on a section of difference compensation fiber (DCF). The DCF with a length of 30 m, 200 m away from the front end of the sensing fiber, is placed in the thermostatic bath (Tmb) with a reference temperature to compensate the unstable output of optical power. Suppose $L_{0}$ ($L_{0}<L$) is a point in DCF, because the time interval of signal acquisition between the point $L_{0}$ and the point *L* is short, it can be considered that the optical power remains constant during this signal acquisition time. In the calibration stage, the temperature of the DCF is set to $T_{r0}$ via Tmb, the intensity of anti-Stokes Raman backscattered light at the point of $L_{0}$ can be expressed as:

(7) $$F(T_{r0},L_{0})=K_{a}\cdot \nu_{a}^{4}\cdot S\cdot P_{0}\cdot \exp[-(\alpha_{0}+\alpha_{a})L_{0}]\cdot R_{a}(T_{r0})$$

In the measurement stage, the temperature of the DCF is set to $T_{r}$, the intensity of anti-Stokes Raman backscattered light at the point of $L_{0}$ can be expressed as:

(8) $$F(T_{r},L_{0})=K_{a}\cdot \nu_{a}^{4}\cdot S\cdot P_{1}\cdot \exp[-(\alpha_{0}+\alpha_{a})L_{0}]\cdot R_{a}(T_{r})$$

Then the ratio of optical power fluctuations during the calibration stage and the measurement stage can be obtained by Equations (7) and (8):

(9) $$P_{1}/P_{0}=[F(T_{r},L_{0})/F(T_{r0},L_{0})][R_{a}\left(T_{r0}\right)/R_{a}(T_{r})]$$

Taking Equation (9) into Equation (3), the temperature at the point of *L* along the fiber is only modulated by Raman scattering light $F(T_{0},L)$ and F(T,L), which can be expressed as:

(10) $$T\left(L\right)=\frac{h\Delta \nu /k}{\ln\left\{1+M\left[\exp\left(h\Delta \nu /kT_{r}\right)-1/\exp\left(h\Delta \nu /kT_{r0}\right)-1\right]\left[\exp\left(h\Delta \nu /kT_{0}\right)-1\right]\right\}}$$

where *M* is the value obtained by the data acquisition card, and *M* can be expressed as:

(11) $$M=\left[F(T_{r},L_{0}\right)/F(T_{r0},L_{0})]\left[F(T_{0},L\right)/F(T,L)]$$

### 2.2. Visual Localization Technology for Tunnel Fire Detection

Optic fiber sensor monitoring physical quantities by laying the fiber in the relevant facility. For the complex distribution of the fiber, or the fiber layout there is a local bending phenomenon. It is difficult for conventional 2D display method to quickly locate the temperature rapid change regions into the actual 3D space coordinate. The visual localization technology can realize 3D presentation of spatial position, and display relative position and associate data in all directions and at multiple angles. The visual localization technology for tunnel fire detection is implemented by the following steps.

#### 2.2.1. Construction of Visual Tunnel Model and Fiber Layout Method

We used the mechanical design software solidworks (SW) to build the visual tunnel model. SW divided the tunnel into two separate entities, tunnel entirety and surrounding rock. The final visual tunnel model is obtained by assembling the two parts in SW. The visual tunnel model we established is single-hole and a two-lane road.

**Figure 2:**
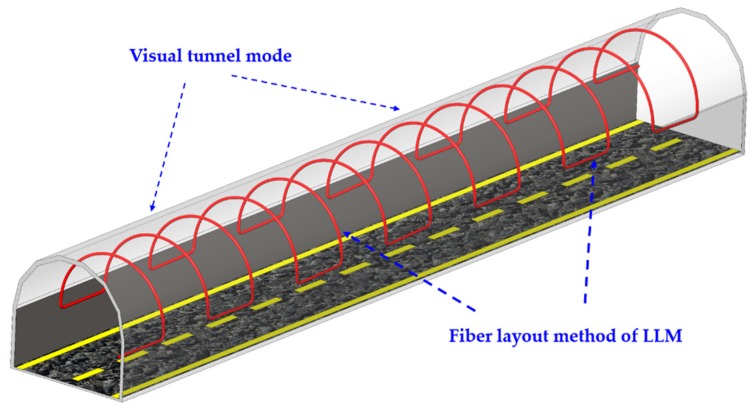
Visual tunnel model and the longitudinal lining model for fiber layout in tunnel.

In order to achieve the fire location and temperature monitoring in the tunnel, we have developed visual localization technology for tunnel fire detection, which is based on the fiber layout method of longitudinal lining model (LLM). The LLM was installed in both longitudinal direction and cross direction. This fiber layout method not only improves the quality of data but also does not influence the overall system performance even if one of the fibers is broken [25,26]. The LLM is obtained by scanning the longitudinal lining curve (LLC). The LLC consists of two parts: the longitudinal curve and the lining curve. The longitudinal curve is distributed orderly in the left and right sides of the tunnel; it is used to monitor the distribution of temperature field in the tunnel. The lining curve adopts an arc-shaped layout and closely follows the tunnel lining structure. Considering that, when a fire occurs in the tunnel, the heat is generated by the fire spreading upwards, and the longitudinal curve is insensitive to fire monitoring. The unique layout of the lining curve is sensitive to vertical fire heat. The LLM not only visually displays the layout status of the fiber in the tunnel, but also displays the temperature along the fiber in real time, and distinguishes the temperature rapid regions by different warning colors. Figure 2 shows the visual tunnel model and the fiber layout method of LLM in the tunnel.

#### 2.2.2. Visual Localization Technology

Visual localization technology is achieved by means of sensor mapping functions in virtual instrument software LabVIEW. We saved the LLM established in Section 2.2.1 as the format that can be recognized by LabVIEW. The format contains the normal vector coordinate information of the model. The normal vector coordinate information is used to display whether the fiber model is selected or not.

In order to map the 2D temperature along the fiber to the visual model of LLM, we have written a sensor layout program. The program is used in deploying virtual temperature sensors on the LLM and mapping the temperature collected by the data acquisition card to the virtual temperature sensors. By reading the vertex array on the LLM according to the distribution direction of the fiber, and inputting the read vertex array into the virtual instrument LabVIEW self-contained programming function index array, thus, the virtual temperature sensors are placed on the LLM.

The unknown points of temperature between the virtual temperature sensors is interpolated by Equation (12). The coordinate value of the unknown points is read by the sensor layout program, and the unknown points weight is given according to the distance between the unknown points and virtual temperature sensors. A smaller weight value is assigned to an unknown point that is closer to the virtual temperature sensors, and the farthest point is given a large weigh value.

**Figure 3:**
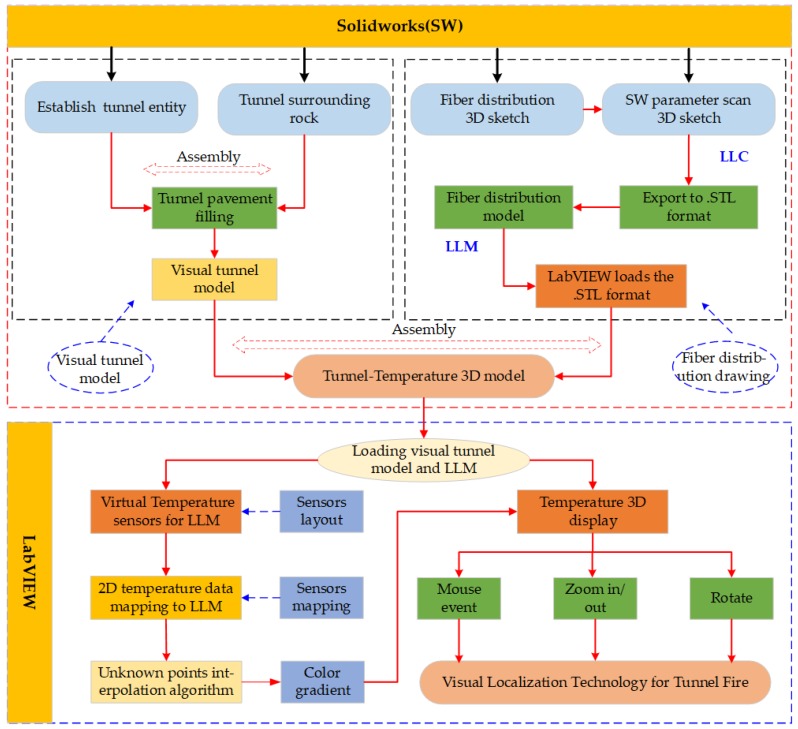
The flow chart of visual localization technology for tunnel fire.

The interpolation algorithm as follows:

(12) $$G(l)=\sum_{i=1}^{m}\frac{W_{i}}{{\left[d_{i}(x,y)\right]}^{r}}/\left(\sum_{i=1}^{m}\frac{1}{{\left[d_{i}(x,y)\right]}^{r}}\right)$$

where G(l) represents the attribution of the unknown point G, and m represents the number of observed points, $W_{i}$ represents the attribute value of the i-th observed point, $d_{i}(x,y)$ represent the distance from the i-th point to the point of G. The interpolation factor r determines the smoothness of the interpolation result, and the larger the r value, the gentler the interpolation result is. Figure 3 shows the flow chart of visual localization technology.

## 3. Experimental Setup

**Figure 4:**
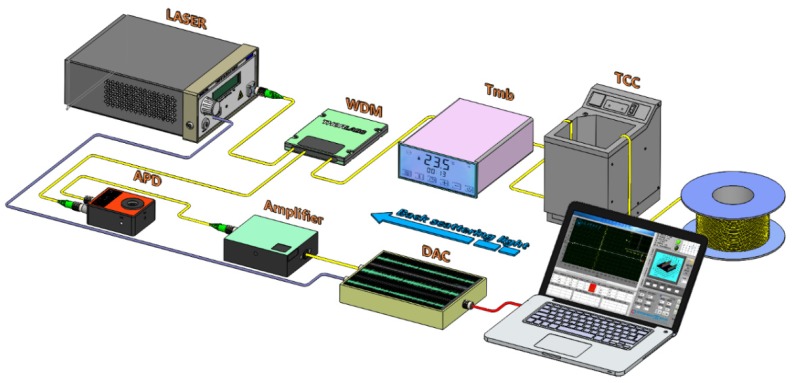
Experimental setup of Raman distributed temperature sensor (RDTS) systems, Tmb: thermostatic bath, WDM: wavelength division multiplexer, APD: avalanche photodiode, DAC: data acquisition card, TCC: temperature control chamber.

The experimental setup shown in Figure 4 has been used to validate the proposed optical dynamic difference compensation algorithm for RDTS systems. The RDTS systems consist of the laser, WDM, Tmb (HZBOHO, BH8001), TCC (BiLon, GDH-3030), APD, DAC, amplifier and the multimode fiber (MMF, 62.5/125 μm). The MMF consists of three sets of fiber under test (FUT), including FUT 1(~20 m) at 1.14 km, FUT 2(~30 m) at 9.6 km, and FUT 3(~50 m) at 18.27 km. The peak power of the laser output is 30 W, operating at about 1550 nm wavelength with a pulse width of 10 ns and a repetition rate of 3 kHz. The pulse light generated by the laser launches into sensing fiber, Raman backscattered light guides it back to the front end of the sensing fiber. APD captures the anti-Stokes light from WDM and converts it to electrical signals, then the electrical signals are averaged 20,000 times by DAC. Then the temperature information along the fiber is demodulated. Table 1 shows the parameters of each device in the experiment.

## 4. Results and Discussion

### 4.1. Optical Dynamic Difference Compensation Improves Temperature Measurement Accuracy

**Figure 5:**
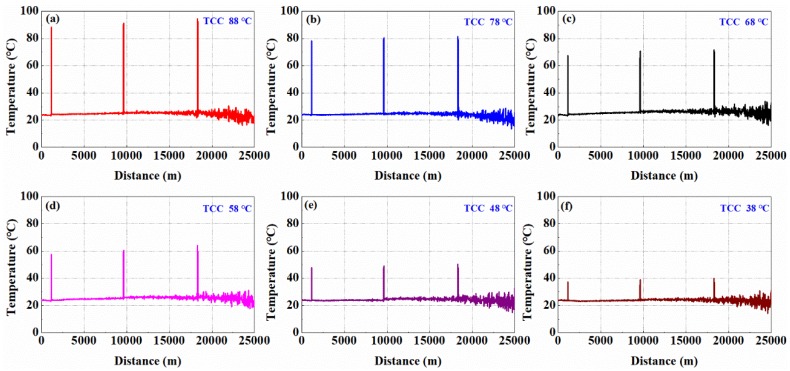
The results of temperature-distance traces along the sensing fiber of 25 km from 38 °C to 88 °C in Fiber Under Test (FUT). (**a**) Temperature-distance traces for FUT at 88 °C; (**b**) Temperature-distance traces for FUT at 78 °C; (**c**) Temperature-distance traces for FUT at 68 °C; (**d**) Temperature-distance traces for FUT at 58 °C; (**e**) Temperature-distance traces for FUT at 48 °C; (**f**) Temperature-distance traces for FUT at 38 °C.

In order to verify the feasibility of the proposed optical dynamic difference compensation algorithm, the temperature measurement experiment was carried out at room temperature around 24 °C. The sensing fiber with a length of 25 km was used. The temperature of FUT was achieved by adjusting the temperature of the water in the TCC. The temperature of the FUT was set at 88 °C, 78 °C, 68 °C, 58 °C, 48 °C and 38 °C, respectively. Figure 5a–f show the temperature-distance traces obtained by the proposed temperature demodulation method of optical dynamic difference compensation algorithm.

**Figure 6:**
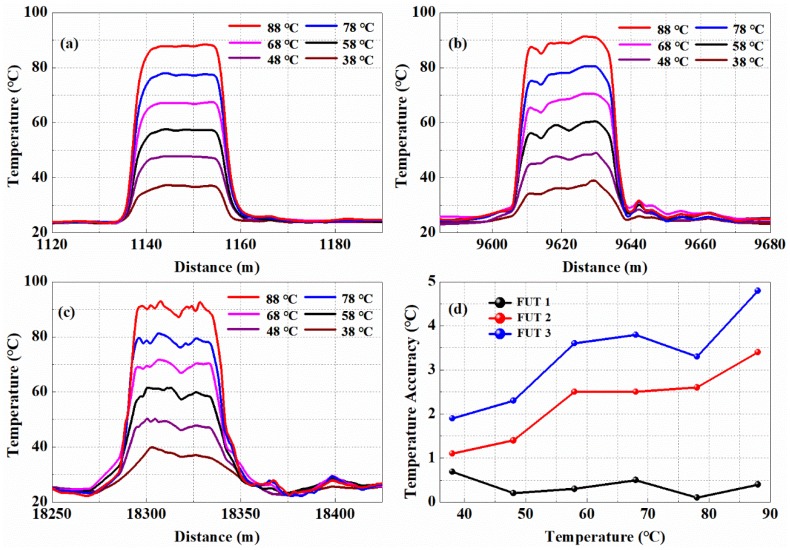
(**a**) Results of temperature measurement in detail of FUT 1; (**b**) Results of temperature measurement in detail of FUT 2; (**c**) Results of temperature measurement in detail of FUT 3; (**d**) Results of temperature measurement accuracy of FUT 1, FUT 2, and FUT 3.

Figure 6a–c show the temperature traces of detection area at sensing section of FUT 1, FUT 2, and FUT 3. Figure 6d shows results of temperature measurement accuracy of FUT 1, FUT 2, and FUT 3. It can be seen from Figure 6d, when the temperature of the FUT is 88 °C, the temperature measurement error is 4.8 °C at the sensing distance of 18.27 km.

**Figure 7:**
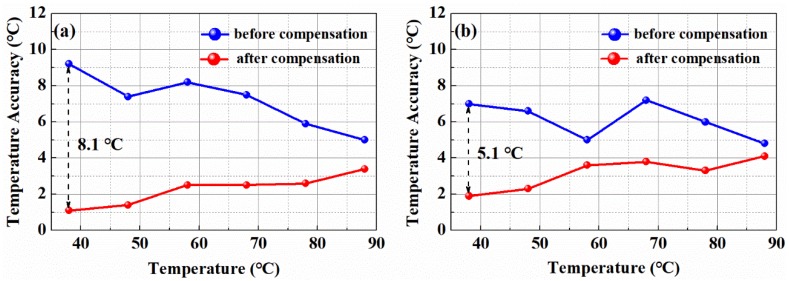
(**a**) Temperature measurement accuracy before and after optical power compensation at FUT 2 (9.6 km); (**b**) Temperature measurement accuracy before and after optical power compensation at FUT 3 (18.27 km).

A contrast experiment by using the convention temperature demodulation method of Equation (3) and the proposed temperature demodulation method of Equation (10) was conducted. Figure 7a,b show the temperature measurement accuracy from 38 °C to 88 °C before and after the optical power fluctuation compensation at the sensing section of FUT 2 and FUT 3. The blue curve and the red curve represent the temperature measurement accuracy before and after the optical power compensation, respectively. After compensating the optical power, the temperature measurement accuracy is optimized from 9.2 °C to 1.1 °C and from 7.0 °C to 1.9 °C at the sensing distance of 9.6 km and 18.27 km, respectively. It is obvious that the optical dynamic difference compensation algorithm can provide a reliable and high temperature measurement accuracy. Moreover, this method is much simpler and more precise to compensate for the optical power fluctuation to improve temperature measurement accuracy at every time.

**Figure 8:**
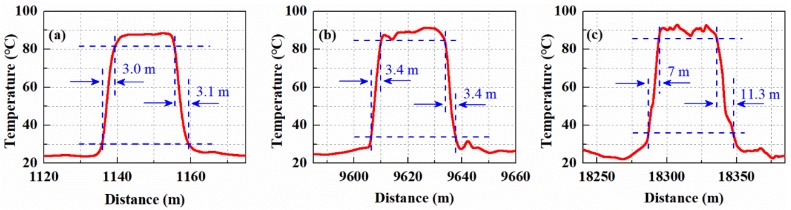
(**a**) Spatial resolution at 1.14 km; (**b**) Spatial resolution at 9.6 km; (**c**) Spatial resolution at 18.27 km.

The spatial resolution (response distance corresponding to 10%–90% temperature step) of the RDTS systems determine location accuracy of a tunnel fire. At the temperature of 88 °C for FUT, the RDTS systems spatial resolution experiment was conducted. Figure 8a–c show the spatial resolution at the sensing distance of 1.14 km, 9.6 km and 18.27 km. It can be seen that the spatial resolution of the systems is 3.05 m, 3.4 m and 9.15 m at the sensing distance of 1.14 km, 9.6 km and 18.27 km, respectively.

### 4.2. Simulation Results of Visual Localization Technology for Tunnel Fire Detection

**Figure 9:**
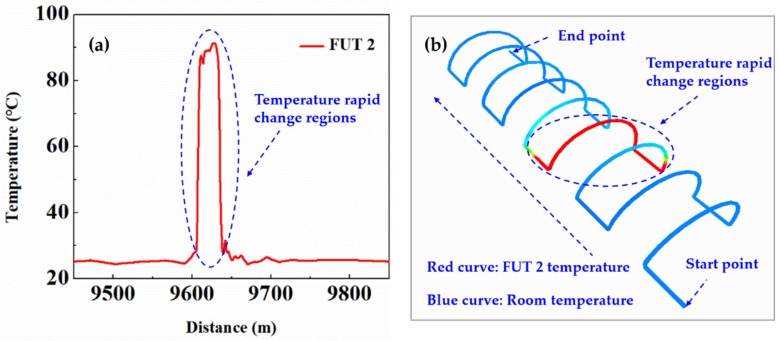
(**a**) The temperature trace at the sensing section of FUT 2; (**b**) The mapping results of 2D temperature trace to visual model of the longitudinal lining model (LLM).

Simulation experiments were carried out to validate the feasibility of visual localization technology. The fiber layout method in the tunnel is in accordance with the visual mode of LLM, and the length of LLM is 212 m. Each group of longitudinal curve and lining curve with a length of 5 m and 18 m, respectively. The LLM is drawn according to the actual ratio of 1:100. Figure 9a shows the temperature trace at the sensing section of FUT 2. The temperature is mapped from 9540 m to 9752 m to the visual model of the LLM. Figure 9b shows the mapping results by sensor layout program. The blue region represents the room temperature along the fiber, the red region represents the temperature rapid change regions along the fiber, namely the temperature at FUT 2. Consequently, assuming that there is a fire in the tunnel, this visual localization technology can locate the fire source to the 3D geographic coordinates of the tunnel. Moreover, combined with the proposed method of optical dynamic difference compensation algorithm, it can achieve rapid positioning of fire source for long-distance tunnel or tunnel groups.

**Figure 10:**
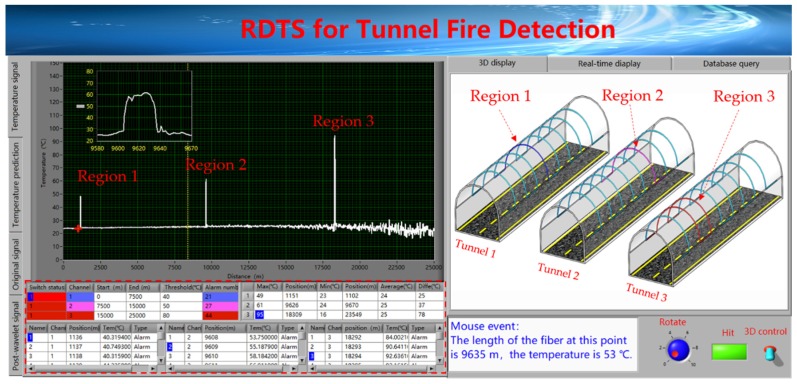
The platform of visual localization for tunnel groups.

Figure 10 shows the platform of visual localization for tunnel fire detection. The sensing fiber is divided into three channels, namely channel 1 (0 m–7500 m), channel 2 (7500 m–15,000 m) and channel 3 (15,000 m–25,000 m). The alarm is set at threshold of these three channels to 40 °C (blue warning), 50 °C (orange warning), and 80 °C (red warning), respectively. The red dotted box shows the warning information of the 2D curve, which includes the number of warning temperatures in each channel, as well as the warning position, the maximum temperature, minimum temperature and the average temperature along the channel, etc. The temperature rapid region 1, the region 2 and the region 3 in the 2D curve are mapped to the tunnel 1, the tunnel 2 and the tunnel 3 in the tunnel groups, respectively. In the tunnel groups, different alarm thresholds are displayed in different warning colors. Blue warning, orange warning and red warning represent alarm thresholds of 40 °C, 50 °C, and 80 °C, respectively. By combining the 2D curve’s warning information with visual localization technology of 3D temperature display, it is possible to quickly and accurately locate temperature rapid change regions.

## 5. Conclusions

We propose and experimentally demonstrate a novel method for tunnel fire detection, which includes optical dynamic difference compensation algorithm and visual localization technology. Experimental results indicate that the temperature measurement accuracy is optimized from 7.0 °C to 1.9 °C at the sensing distance of 18.27 km by using the optical dynamic difference compensation algorithm. Simulation results show that the visual localization technology can quickly locate 2D temperature rapid change regions into the actual 3D space environment. This paper is expected to achieve accurate monitoring as well as visual localization of tunnel fire.

## Figures and Tables

**Table 1:** Parameters of each device in the experiment.

Key Device	Performance Parameters
Laser	Center wavelength: 1550.1 nm, Pulse width 10 ns
APD	Response range: 900–1700 nm
WDM	Operating wavelength: 1450 nm/1550 nm/1663 nm
Amplifier	Bandwidth: 50 MHz
DAC	4 channels, 8 bit, 50 M/s
Tmb	temperature range: −30 to +100 °C/0.1 °C
TCC	Temperature fluctuation: ±0.1 °C
